# White butterflies as solar photovoltaic concentrators

**DOI:** 10.1038/srep12267

**Published:** 2015-07-31

**Authors:** Katie Shanks, S. Senthilarasu, Richard H. ffrench-Constant, Tapas K. Mallick

**Affiliations:** 1 Environment and Sustainability Institute, Biosciences, University of Exeter, Falmouth, TR10 9FE, UK; 2 Centre for Ecology and Conservation, Biosciences, University of Exeter, Falmouth, TR10 9FE, UK

## Abstract

Man’s harvesting of photovoltaic energy requires the deployment of extensive arrays of solar panels. To improve both the gathering of thermal and photovoltaic energy from the sun we have examined the concept of biomimicry in white butterflies of the family Pieridae. We tested the hypothesis that the V-shaped posture of basking white butterflies mimics the V-trough concentrator which is designed to increase solar input to photovoltaic cells. These solar concentrators improve harvesting efficiency but are both heavy and bulky, severely limiting their deployment. Here, we show that the attachment of butterfly wings to a solar cell increases its output power by 42.3%, proving that the wings are indeed highly reflective. Importantly, and relative to current concentrators, the wings improve the power to weight ratio of the overall structure 17-fold, vastly expanding their potential application. Moreover, a single mono-layer of scale cells removed from the butterflies’ wings maintained this high reflectivity showing that a single layer of scale cell-like structures can also form a useful coating. As predicted, the wings increased the temperature of the butterflies’ thorax dramatically, showing that the V-shaped basking posture of white butterflies has indeed evolved to increase the temperature of their flight muscles prior to take-off.

Solar concentrators use mirrors and lenses to capture light and direct it towards smaller areas of photovoltaic (PV) material where the solar energy is converted into electricity^1^. In this way the cost of the overall system is reduced by decreasing the area of photovoltaic material required which is typically the most expensive part of a PV solar panel^1^,^2^. However, the introduction of these optical devices to focus light onto these solar cell(s) can result in very bulky systems. Although solar concentrators can reduce solar energy costs and improve efficiencies, their weight and size therefore often limits their deployment^3^,^4^. Current solar concentrators vary widely in design and even the simple polishing of metal can result in a reflective mirror finish but such polished surfaces are very heavy and specific curved shapes are difficult and therefore expensive to manufacture^5^,^6^. Reflective film adhered to plastic mirrors is a second option but this setup often has low reflectivity when applied to complex surfaces^6^. Polymer mirror films are a more recent third method to gain reflectance values of >90% but require specially designed structures to gain the appropriate shapes for a given application^7^,^8^. Vacuum metalizing is therefore the current best option but this process is highly dependent on the material and surface quality it is bonded with in order to ensure a high quality mirror finish^5^,^9^. Given the limitations of all existing systems, further study into possible lightweight reflective materials and structures is important. The benefits of a lightweight, easily applied reflective material or coating would not only improve the development of solar concentrator technologies but may also be beneficial to many other disciplines where lightweight highly reflective coatings are desirable.

The white butterflies of the genus *Pieris* take flight before other butterflies on cloudy days when solar inputs to flight muscle warming are limited. This ability to heat up quickly on cloudy days has been anecdotally suggested to relate to the V-shaped posture they adopt whilst basking in cloudy conditions, a process we here term ‘reflectance basking’. These white butterflies do indeed show high wing reflectance based upon a unique display of pterin containing nano-beads within their individual wing scales as extensively reported by Stavenga *et al*.^10^,^11^,^12^, Giraldo *et al*.^13^,^14^ and Morehosue *et al*.^15^. Luke *et al*.^16^ expand on this descriptive work by removing the pterin beads and showing that overall reflectance is decreased by a third in the absence of the beads themselves. The precise arrangement of the pterin beads within the scale cell appears critical as it shows a quasi-random pattern that has recently been proposed to be optimum for efficient light manipulation^17^.

Here we therefore investigate if the wings, or some derivation thereof, of the white *Pieris* butterflies can be used to develop a novel, lightweight reflective material directly applicable to solar concentrators. To investigate if a consideration of the photonics of butterfly wings is indeed useful in solar concentrator design we chose to first answer five specific questions. First, can we prove practically that the butterflies concentrate light, and indeed heat, onto their thorax? Second, is there an optimum angle with which they accomplish this and which we would therefore have to adhere to in solar concentrator design? Third, does the light reflected by the butterfly wings themselves actually match the input requirements of any given photovoltaic solar cell? Fourth, can whole butterfly wings thus be used directly to increase the output from a given solar cell? Finally, can specific sub-structures from the wing (e.g. a mono-layer of removed scale cells) or bead-like coatings (e.g. a coating of nano-beads with the same orientation and properties of the pterin beads) be used to achieve similarly improved solar cell outputs?

Butterfly wings are in fact surprisingly complex as butterflies not only have pairs of wings that are effectively linked in flight (and overlap at rest) but the scale cells on their wings also show dramatically different morphologies and orientations. Further, these scale cells can exist as complex overlapping layers therefore potentially conferring complex overall optical properties on the whole wing, as detailed extensively by the work of Vukusic *et al*.^18^,^19^,^20^ and also by Kolle *et al*.^21^. Such complex naturally occurring structures can be used for various modern applications in a process known as ‘biomicry’^22^,^23^,^24^, however no studies have yet examined the *Pieris* wing structures as a basis for reflective materials in solar photovoltaic concentrators. Johnsen and Widder^25^ showed that the pterin bead size is optimized for light scattering and that the two types of wing scales (‘cover’ and ‘ground’ scales) together produce wide-angle scattered light. Stavenga and co-workers also argue that to gain the full reflectance from the pierid wing a complete model including all components of the wing structure would be required. This would initially suggest that a single layer of scale cells or a thin coating of nano-beads correctly orientated would have insufficient optical performance to enhance inputs to a solar cell. One of the central aims of the research described here was therefore to see if a mono-layer of scale cells could recapitulate the reflective properties of the whole wing. Surprisingly, here we show that wings from the large white butterfly do indeed increase the efficiency of photovoltaic cells when the wings are held at a critical optimal angle for the concentration of both heat and light. Further, this whole wing configuration not only dramatically increases the power to weight ratio of the butterfly-solar cell structure but critically similar reflective properties can be achieved from a single mono-layer of removed scale cells. This work suggests that scale cell-like structures or indeed just coatings of correctly oriented nano-beads may be useful in even more lightweight coatings.

## Results

### Parallels between the V-shape of a basking butterfly and the V-trough concentrator

**Figure 1:**
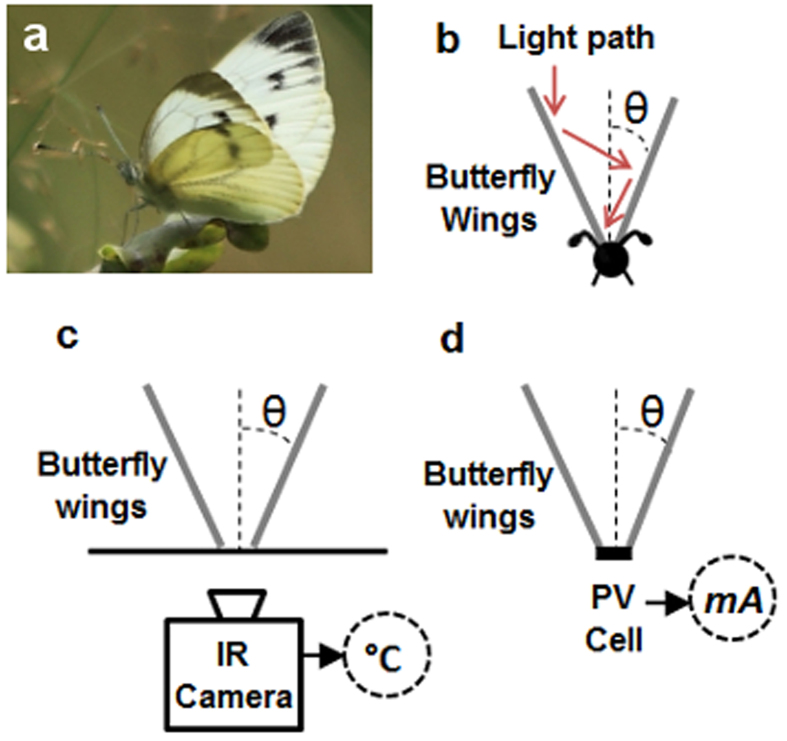
White butterflies as solar concentrators. (**a)**, Photograph of large white (taken by Richard ffrench-Constant) with wings in ‘V-shape’ basking posture. (**b)**, Schematic diagram of theoretical light concentration towards thorax via reflection from wings of butterfly. (**c)**, Method for measuring wing angle effect on ‘body’ temperature (°C). (**d)**, Method for measuring wing angle effect on current output (mA) from solar cell in place of ‘body’.

As white butterflies of the family Pieridae are especially effective at early take-off on cloudy days, and can therefore fly before other groups of butterflies in poor weather, we reasoned that this ability is due to the V-shaped posture they adopt with their wings while ‘thermal’ basking (Fig. 1a). This V-shaped posture should gather and focus solar energy, reflected by the wings, onto the body (thorax) of the butterfly (Fig. 1b) and thus, increase the temperature of the flight muscles prior to take-off. The V-shaped design of the butterfly is therefore strikingly similar to the V-trough solar concentrator which uses mirrored side walls to focus light towards a small area of photovoltaic material^3^,^26^ (Fig. 1d) thereby increasing the output power of any solar cell to which it is attached^4^,^27^.

### Determining the optimal angle at which a butterfly should hold its wings

**Figure 2:**
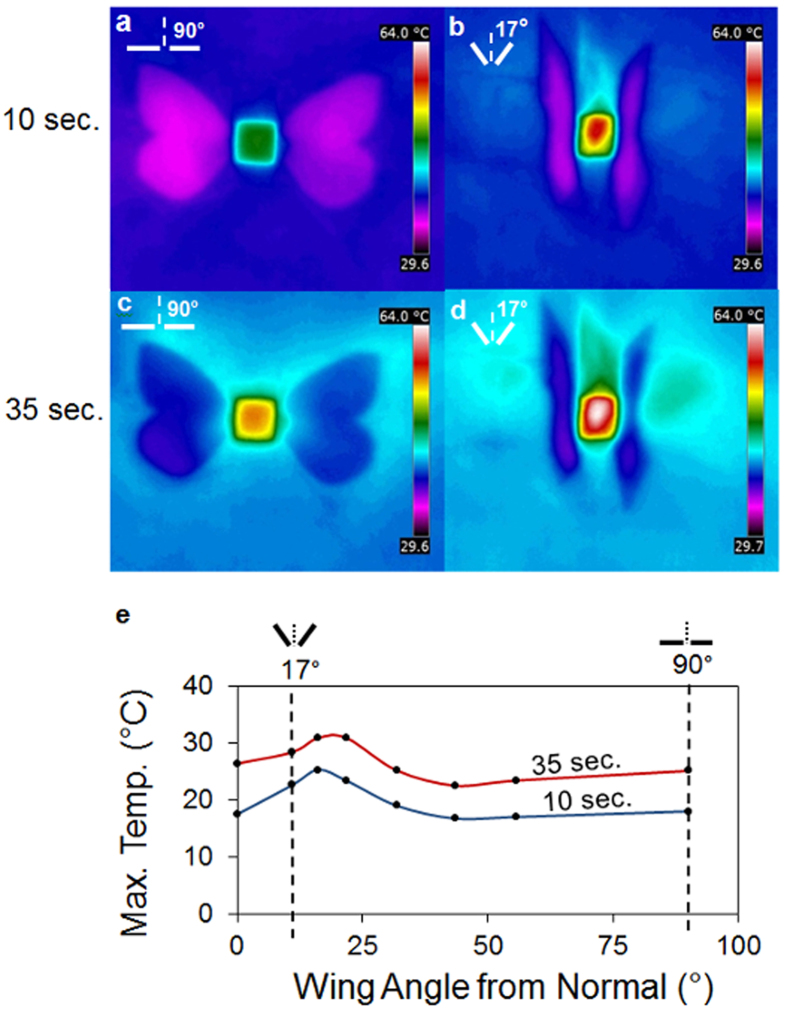
Thermal analysis of butterflies with wings held open (90°) or in a V-shape (17°). (**a**,**b**) Increase in temperature seen following 10 second exposure to one sun equivalent. (**c**,**d**) 35 second exposure to one sun equivalent. Note the dramatic increase in temperature at the equivalent location of the thorax when the wings are held at the optimal basking angle of 17°. (**e**), Graph of ‘body’ temperature as a function of wing angle for two sunlight exposure times of 10 seconds and 35 seconds.

To directly test the hypothesis that the butterfly uses its wings to increase the temperature of its body and to determine the optimal angle at which the wings should be deployed, we measured the temperature of the butterflies’ ‘thorax’ (at an equivalent position between the open wings) using an infra-red camera (Fig. 1c). Following 10 and 35 second exposure to the equivalent of ‘one sun’ (light from an artificial source mimicking bright sunlight^28^) we measured the temperature of the butterfly ‘thorax’ at different wing angles (measured from the vertical or normal). Using this experimental set-up we found that 17° from normal was the optimal angle for the butterfly to hold its wings and that this increased the ‘body’ temperature by 7.3 °C more than the temperature achieved when the wings were held flat (at 90°) (Fig. 2). These observations support the concept that thermal basking does indeed increase the temperature of the butterfly body and therefore directly implies that a similar experimental design could be useful in improving solar inputs to photovoltaic cells.

### Matching the input requirements of a solar cell using different butterfly species

**Figure 3:**
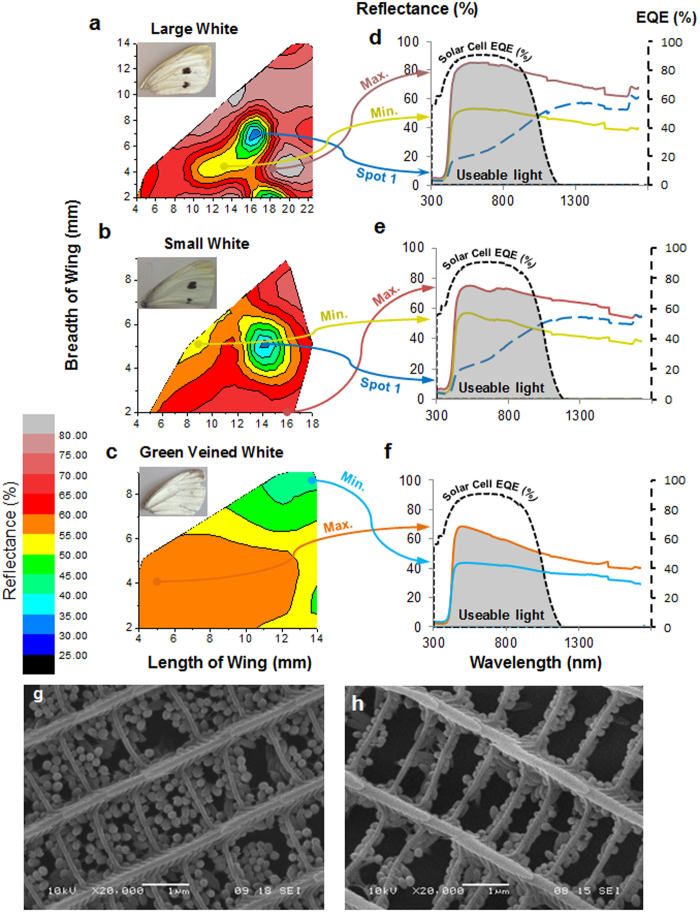
Mapping reflectance across the butterfly wing. Average percentage reflectance map for wings of the large (**a**), small (**b**) and green-veined white (**c**) butterflies. Insets show how each wing appears in normal daylight. (**d,e,f)** Reflectance spectrum for specific notable areas (maximum, minimum and black spot areas). Note how reflectance decreases dramatically over the black ‘spots’ present of the forewing of females of the large (**d**) and small white (**e**) whose black scales lack the reflecting pigment containing beads, see text for discussion. (**g)**, SEM of wing scale containing packed pterin beads. (**h)**, SEM of black spot area of wing scale containing significantly less pterin beads.

Before we could test this exciting hypothesis, we first needed to match the wavelength range reflected by the butterfly wings to the input requirements of a given solar cell. There have been various studies into how natural structures can affect light^18^,^19^,^20^,^21^ and butterfly wing structures in particular are well researched^12^,^29^,^30^,^31^,^32^,^33^ but none of this prior work relates specifically to solar cells. To determine which wings were best matched to a specific solar cell type, we first mapped the reflectance patterns across the forewings of three common *Pieris* species the large white, *P. brassicae*, the small white, *P. rapae*, and the green-veined white, *P. napi* (Fig. 3a–c). These reflectance maps strikingly emphasise the contrast between the low reflectance associated with the black spots present on the butterfly forewings (Fig. 3a,b) with the high reflectance of the surrounding white areas^18^,^21^. These gradients in reflectance across the wing are explained by well-known differences in the ultrastructure of black and white wing scales in this group^10^,^11^,^13^,^15^,^34^,^35^. In the white wing scales the scale windows (gaps in the scale structure) are partially filled with ovoid shaped granules or ‘beads’ (Fig. 3g). These ovoid beads contain the white pigment pterin which absorbs light in the short-wavelength range but strongly scatters light outside the pigment absorption range^11^,^13^,^34^,^35^,^36^. The black scales located in the two black spots lack these pigment carrying beads (Fig. 3h) and the black pigment melanin, which has a broad absorption spectrum, located in the cross-ribs of the scale itself^13^,^34^.

### Using the wings of the large white butterfly to increase power output from a solar cell

**Figure 4:**
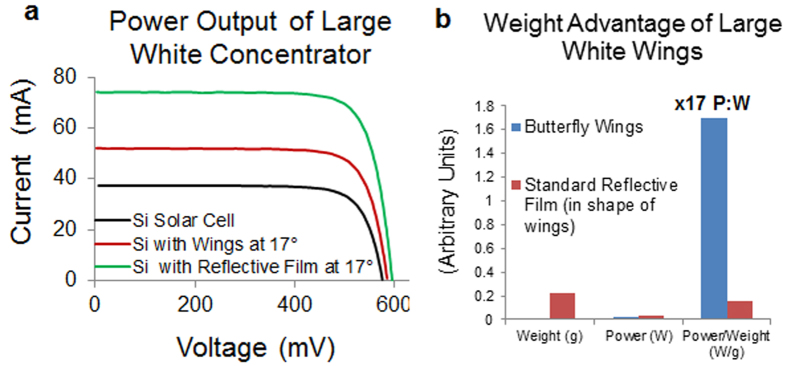
Butterfly wings increase both the output power and the final power to weight ratio of solar cells. (**a)**, Power output of a mono-crystalline silicon (Si) solar cell either alone, or with large white wings versus reflective film held at the optimal angle of 17°. (**b)**, Histogram representing the relative changes in both power, weight and the subsequent power to weight ratio of large white butterfly wings versus reflective film.

The highest reflectance came from the forewings of the large white butterfly and this reflectance was also well matched to the input requirements of a mono-crystalline silicon cell (average of 78.9% reflectance over 400–950 nm range, Fig. 3a). We therefore attached the wings of the large white butterfly to a 1 cm × 1 cm mono-crystalline silicon solar cell to test for any increase in output power. Attaching the wings increased the maximum power by 42.3% (from 16.8 mW to 23.9 mW) and when compared to the weight of standard reflective film increased the power to weight ratio 17 fold (Fig. 4a,b). Moreover, a mono-layer of scale cells removed from the wing onto adhesive tape also maintained similar high reflectance properties (62% reflectance from 400–950 nm, maintaining 78.6% of the original reflectance). Suggesting that only a single layer of scale cells is necessary to generate high levels of reflectance, rather than a complex multi-layered structure as found in the wing itself^18^,^25^ and therefore directly increasing their potential utility in making a reflective coating.

## Discussion

The V-shaped reflectance basking of the family Pieridae is most definitely comparable to V-trough solar concentrators and even more so when considering studies into the segmented surface structure of solar concentrators as carried out by Zanganeh *et al*.^8^, Nilsson *et al*.^26^ and more broadly by Sangster *et al*.^37^. Nilsson and co-workers prove that the introduction of micro-structures onto the surface of reflectors has many benefits including a more uniform distribution of light upon the receiver and higher acceptance angles. The wings of the pieridae have a similar micro structure upon their wings due to the ‘tiling’ of their scales. Both of the aforementioned benefits reported by Nilsson *et al*. may be crucial to the pierid butterflies when basking in overcast diffused light conditions (light is incident from all angles, not just directly from the sun). Further investigation into the acceptance angle of these basking butterfly wings is however required.

In our study the optimum wing angle for light concentration by the butterfly wings was found to be 17° for both the thermal and photovoltaic receiver conditions. This angle does not however indicate with certainty the exact angle with which real butterflies will position their wings due to slight differences in the geometry of our set-up and that of a real butterfly. Specifically, the receiver size and shape in the tested case was a 10 mm by 10 mm square solar cell instead of the thorax/flight muscle area of the butterflies which is in the range of 2 to 3 mm by up to 20 mm (large white). The optimum angle in V-trough concentrators can be influenced by the receiver shape and size, the reflective mirror heights, location (i.e. latitude and longitude of the place) and the solar tracking method used^38^,^39^,^40^. In the case of the pierids, the surface structure of their wings as well as the shape and size of their target area (flight muscles) will predominantly decide the angle with which their wings are held. Other factors however could include: the desired energy/temperature upon flight muscles^41^; the time of year (sun’s location, ambient temperature, thorax size^42^); and location (global horizontal irradiance values)^38^,^43^. This optimum angle does however prove that other receiver dimensions and applications are possible with these wings and that they are not solely optimised for the characteristics of the thorax.

The excellent match between the reflectance spectra of the large white butterfly and the working range of a monocrystalline silicon solar cell ensures that useful light rays are incident upon the solar cell. The butterflies’ main aim in reflectance basking is to heat their flight muscles^41^ whereas photovoltaic solar cells work less efficiently when heated^44^ and so it would have been feasible that the reflected wavelengths would only be harmful IR wavelengths. The reflectance spectra given in Fig. 3a however reassure the use of wavelengths in the 450–950 nm range. These results would indicate that if used in larger concentration systems (500 fold concentration) that receiver cooling would be required to avoid damage to the photovoltaic receiver. This is a common necessity for current concentrator technology at high concentration ratios^44^,^45^.

The I-V output curves show a 42.3% increase in power from the solar cell with attached large white butterfly wings. In terms of increased solar input (solar concentration) this works out as a concentrating effect of 1.3x, compared to the 2x concentration achieved by the reflective film. However in terms of *weight*, the butterfly wings have 17x the power to weight ratio of the reflective film structure. In theory, the maximum concentration ratio possible using the angle of the wings and receiver size with no light loss, would be 7.5 x concentrations. The miss-match in values however is due to the configuration of the wings where most light can be lost to the front and rear where there is no wing coverage. The 2x concentration result from the reflective film wings prove the majority of the loss is due to the wing configuration and not the wings themselves. A different configuration of the wings, with a smaller receiver similar to the butterflies’ thorax should result in even higher I-V values with less loss.

In conclusion, these striking results have several implications both for the biology of butterflies and for the design of more lightweight but efficient solar concentrator systems. First, the infra-red measurements of butterfly body temperature confirm the assumption that the thermal basking exhibited by pierid butterflies really does provide an increase in thorax temperature proving that their V-shaped posture is an effective thermal basking method. Second, butterfly wings are both highly reflective and much lighter than any current reflective material. Mimicking these reflective structures with similar power to weight properties will be extremely useful in the design of new reflective materials for use in applications where weight is a limiting issue, such as flight. Third, and perhaps most obviously, this suggests that butterflies have evolved to concentrate light effectively for their needs and supports the idea that any given problem may first have been solved by nature^22^,^23^,^24^. Finally, despite the apparent complexity of the multi-layered array of butterfly scales on the wing, here we have shown that a simple mono-layer of scale cells removed onto adhesive tape is also highly reflective. Taking this analogy to its logical end point, we further speculate that nano-fabrication of a layer of ovoid pigment containing beads will also form a reflective and light weight mimic of a pierid scale cell, provided that the nano-beads are presented in their correct orientation. Not only could this potentially enhance the properties and application of reflective materials but it could also expand the application of technologies such as solar concentrators which are currently severely limited by power to weight issues.

## Methods

### Light concentration theory

Given that the V-shape is known to concentrate light as long as there is light reflectance and an acute angle between the reflectors then much of the theory of solar concentrators can be applied to the butterflies. The geometric concentration ratio (C) of a solar concentrator can be estimated using Eqn. 1^28^ and hence an estimation of the potential concentration ratio of the butterflies and of a combination of butterfly wings and solar cell can be calculated.


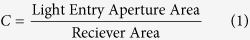



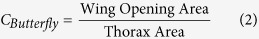



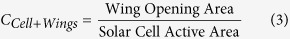


The concentration ratio is a value used to categorise and analyse the efficiency of solar concentrators. The power of light incident on a receiver (P_r_ on butterfly body or solar cell) is dependent upon the optical efficiency (η_o_), concentration ratio (C), incident irradiance on the system (I_i_), and the receiver area (A_r_), such that:


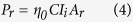


In the case of concentrator photovoltaic systems; where the receiver is 1 or more solar cells, the final power output produced from the solar cell(s) would be the efficiency of the cell multiplied by the power incident on the cell(s), (P_r_). Similarly the term effective concentration ratio should equal the geometrical concentration ratio minus optical and solar cell losses, or in other words, multiplied by their efficiencies^28^ as in Eq.5.


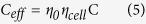


The possible optical losses in the butterfly wing configuration include; light rays incident upon the wings but which are reflected to the front or rear -where there is no wing or body coverage- or even back out the top opening area, and also the efficiency at which the wings reflect the light—the reflectance. Instead of solar cell efficiency, the butterflies presumably would have heat transfer efficiency, dependent on their initial body temperature, incident temperature/energy from sunlight, and the ambient temperature of their environment.

### Measurements Performed

The infra-red (IR) camera shots were taken with a FLIR T425 camera positioned underneath a structure consisting of the butterfly wings separated at the base by 10 mm. The wings were positioned manually at varying angles and exposed to 1000 W/m^2^ of light from a Wacom AAA continuous solar simulator (model: WXS-210S-20, AM1.5G). The optimum performance was found when θ equaled roughly 17°, which was also the optimum angle for power output of the solar cells. This angle was maintained to compare results. The receiver size and shape in the tested case was a 10 mm by 10 mm square solar cell, although this could have been resized to replicate the butterflies body, this would affect the solar cell performance and perhaps not give as promising results as were obtained. Without the replication of the hairy thorax and exact wing positioning (perhaps more cone like than the simple 2D V-shape) the prediction of the butterfly basking wing angle would still not be 100% accurate. The exposure time under the Wacom AAA continuous solar simulator (model: WXS-210S-20, AM1.5G) set at 1000 W/m^2^ was noted and IR images recorded after 10 and 35 seconds. A cool down period of at least 7 minutes was allowed between each set of measurements and a base measurement was taken before switching on the solar simulator, later subtracted from measurements to reduce error due to ambient temperature changes.

**Figure 5:**
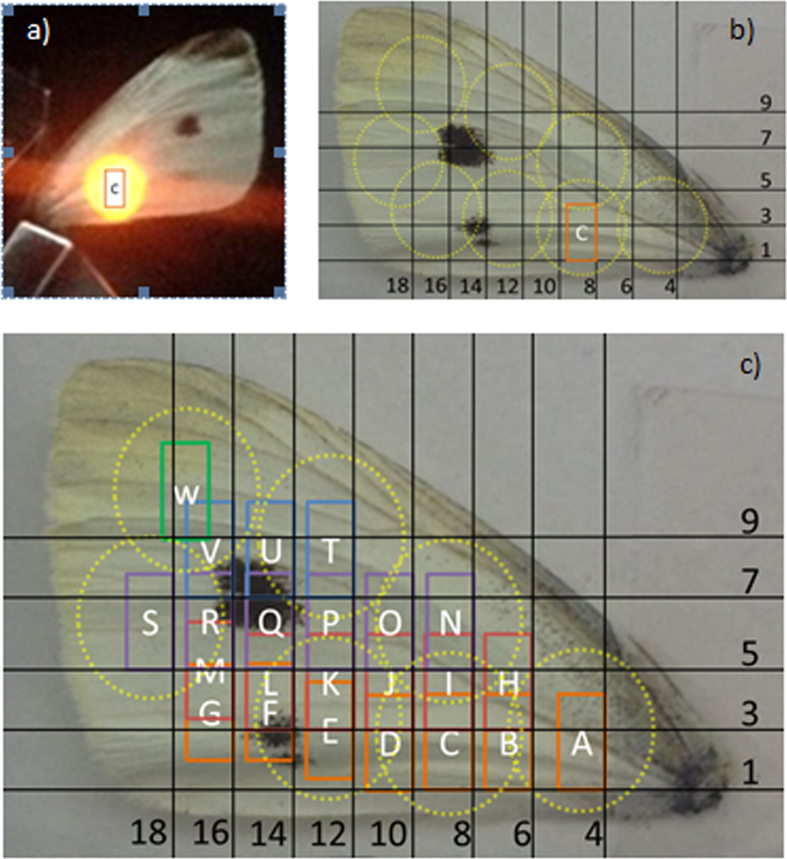
Reflectance mapping method. (**a**), Photograph of forewing placed against integrating sphere at the third measurement position of 8 mm along and 1 mm up (position C). (**b**), An indication of the limit of measurements due to porthole size represented by the yellow dotted circles. (**c**), The full mapping technique with position labels. The exact location of incident beam is not known but confined to the rectangles labelled alphabetically in order of measurements taken.

The reflectance was measured over the wavelength range of 300–1750 nm using a Bentham PVE300 system, the maximum and minimum reflectance spectra are shown in Fig. 3 for the small white, large white and green veined butterfly wing samples. Within this range a wavelength interval of 5 nm was taken and by moving across the wing (x-axis) and up the wing (y axis) in 2 mm steps, the wing was manually mapped as shown in Fig. 5 b,c. The response of the monocrystalline silicon cell (1 cm × 1 cm) was also measured using the Bentham and the external quantum efficiency (EQE) plotted in Fig. 3 graphs a to c, to show the wavelength compatibility between the reflectance spectra of the butterfly wing and working range of the solar cell.

Due to their promising reflectance results, the large white wings were tested further as reflectors in a V-trough CPV systems with a 10 mm × 10 mm solar cell placed between the wings. Figure 4a shows the results obtained with and without wings as a reflector. Reflective film cut in the same shape as the wings was also measured with the solar cell for comparison. The I–V results were taken of a 10 mm by 10 mm monocrystalline silicon solar cell with the 4 large white wings secured at the base and manually angled under the Wacom AAA continuous solar simulator (model: WXS-210S-20, AM1.5G) at 1000 W/m^2^ and using an EKO MP 160 I-V tracer. The monocrystalline silicon solar cells used were from Narec Solar, now known as Solar Capture Technologies. ‘Standard’ reflective film used was RF-015A from Qingdao Lingding Technology Ltd.

### Performance calculations for butterfly wings as solar photo voltaic concentrators

Using equation (3) the theoretical concentration ratio of the wings at 17 degrees from normal with a 100 mm^2^ receiver area is calculated as 7.5x. In ideal conditions with 100% optical efficiency this would translate into the short circuit current of the solar cell without concentration (1 sun) being increased from 36.985 mA to 277.388 mA with concentration from the wings (7.5 suns). When incorporating reflectance loss, using the average reflectance taken for the large white wing samples over the monocrystalline silicon cells response range, 78.9%, the short circuit current under concentration from the wings should be 218.86 mA but practical measurements gave 52 mA. This mis-match in theoretical to practical values suggests there is a large percentage of light lost to the front and rear of the configuration but again it should be remembered that in the case of the pierids, the receiver size is much smaller and hence there is less space for light rays to be lost to the front or rear.

Using the short circuit results gained from the practical testing and working backwards, the effective concentration ratio of the butterfly wings with the solar cell is 1.3x and with the reflective film cut in the shape of the wings is 2.0x proving the majority of the loss is due to configuration and not the wing’s themselves. A different configuration of the wings, with a smaller receiver similar to the shape and size of the butterfly body should result in higher I-V values with less loss. Solar concentration will increase the receiver (solar cell) temperature which can lead to a decrease in their performance but for low concentration levels (up to 10x) this is not usually, and was not in these experiments, an issue but may need to be considered under longer exposure times.

## Additional Information

**How to cite this article**: Shanks, K. *et al*. White butterflies as solar photovoltaic concentrators. *Sci. Rep*. **5**, 12267; doi: 10.1038/srep12267 (2015).
